# Improving Acceptability and Uptake Behavior for Internet-Based Cognitive-Behavioral Therapy

**DOI:** 10.3389/fdgth.2021.653686

**Published:** 2021-03-25

**Authors:** Anthony Molloy, Donovan M. Ellis, Langting Su, Page L. Anderson

**Affiliations:** Department of Psychology, Georgia State University, Atlanta, GA, United States

**Keywords:** acceptability, internet-based cognitive behavioral therapy, mental health, treatment rationale, financial incentive, digital health, treatment access, uptake

## Abstract

Internet-based cognitive behavioral therapy (iCBT) programs have the potential to improve access to mental healthcare, but they are not viewed as acceptable nor widely utilized by the general public. This study tested whether two acceptance-facilitating interventions improved acceptability and uptake-related behavior for therapist assisted and self-guided iCBT. Participants were randomly assigned to read a treatment rationale for iCBT (vs. a brief definition) and to receive a small financial incentive (or not) for seeking more information about evidence-based iCBT programs. Participants (*N* = 662) were a diverse group recruited from a University participant pool and the surrounding community. Participants completed standardized measures of attitudes toward and outcome expectancy for iCBT and a single question about willingness to use it and were given the opportunity to get information about accessing evidence-based iCBT programs. A series of MANCOVAs showed small, positive effects of the treatment rationale on attitudes and outcome expectancy for both self-guided and therapist-assisted iCBT, but not for willingness to use it. A hierarchical logistic regression model found no effect of the treatment rationale or financial incentive on whether participants sought additional information about how to access iCBT, although psychopathology symptoms and identifying as White or multiracial were positively associated with information-seeking. Inconsistent with past research, participants rated therapist-assisted and self-guided iCBT as equally acceptable. Participants recruited from the community reported greater willingness to use iCBT than University students. These results underscore the urgent need for further research toward improving the acceptability and uptake of iCBT so that it may better fulfill its potential to fill the gap in unmet mental health need.

## Introduction

Internet-based cognitive behavioral therapy (iCBT) programs are cognitive behavioral interventions that treat psychological problems via digital platforms. Internet-based cognitive behavioral therapy programs have been shown to reduce symptoms across a range of mental disorders, including post-traumatic stress disorder (1), social anxiety disorder (2), and panic disorder (3), among others. Internet-based cognitive behavioral therapy creates an opportunity to disseminate treatment to people who cannot access face-to-face therapy, as over half of the global population has access to the Internet (4). Additionally, iCBT programs maintain fidelity with treatment protocols in a way that face-to-face treatment delivery in community settings may not (5). Given the insufficient number of licensed mental healthcare providers in the U.S., particularly in areas like rural communities (6), iCBT represents an opportunity to substantially increase access to evidence-based treatment delivered as intended.

Internet-based cognitive behavioral therapy can include support from a therapist or be delivered in a self-guided format. Therapist-assisted iCBT is thought to increase client adherence and reduce attrition (7). An obvious advantage of self-guided iCBT is that a person does not need to find a therapist to access mental healthcare, but a trade-off is that people using self-guided formats may not engage long enough to benefit as much (or at all). One meta-analysis of 12 randomized controlled trials for iCBT for depression and anxiety found that whereas therapist-assisted iCBT programs demonstrated large effect sizes for treatment outcomes, iCBT programs without therapist guidance or support showed small to moderate effects (8). Overall, people benefit from iCBT when paired with therapist assistance or used alone, although the magnitude of effect is likely higher for programs with therapist assistance (9).

Despite its efficacy, iCBT is widely underutilized by the general public (10–12), perhaps because they do not view it as an acceptable form of mental health treatment. Research in this area has defined and operationalized the concept of “acceptability” for digital mental health interventions in a variety of ways. Overlapping constructs like satisfaction, feasibility, and usability are used interchangeably with acceptability (13). Operational definitions include single Likert scale items that assess participants' willingness to use iCBT (14, 15), longer questionnaires designed for individual studies (16), and one psychometrically validated questionnaire that assesses attitudes toward psychological interventions that are delivered online (17). Studies have also operationalized acceptability using validated self-report measures for other constructs, like outcome expectancy—the expectation that one will benefit from treatment (18). The lack of precision in the conceptualization and measurement of the acceptability of iCBT may explain why estimates of the acceptability of iCBT vary widely across research studies.

People who use either self-guided or therapist-assisted iCBT report a high degree of user satisfaction (19–21). However, large survey studies have found that most people are unfamiliar with digital mental health interventions such as iCBT (14) and that people prefer other forms of treatment over Internet-based therapy (22). Therapist-assisted iCBT programs are generally rated as more acceptable than self-guided programs (23, 24), but one survey study found that only 16% of non-treatment-seeking adults would consider using therapist-assisted iCBT to address a mental health concern (16). The significant contrast between high user satisfaction in treatment studies and low acceptability in the general population may be due to the “denominator problem” (25). This refers to a bias that can result when a large number of people are invited to participate in a treatment study, but only the small proportion of those who are motivated and interested volunteer and enroll. However, even large survey studies that recruit potentially biased samples, such as people seeking treatment on mental health clinic websites, have found low acceptability for iCBT (26, 27). This points to a clear need for strategies to increase iCBT's appeal to potential users.

Treatment rationales, which describe how specific therapy interventions work, have long been shown to improve outcome expectancy for face-to-face psychotherapy (28). A handful of studies have incorporated treatment rationales for digital mental health interventions into video or text-based materials designed to improve acceptability and related constructs. Studies generally find that these acceptability-facilitating interventions improve acceptability and intention to use digital mental health programs (23, 24, 29, 30) but not all (31). One limitation to this literature is that most studies used samples that were small or that may not be representative of the general population: Mitchell and Gordon (24) studied a small (*N* = 20) sample of undergraduate students, Ebert et al. (29) studied primary care patients, and Soucy et al. (30) recruited participants who had already demonstrated an interest in using iCBT. Only one study has examined the impact of an intervention to improve acceptability of both self-guided and therapist-assisted programs (23).

No study to date has examined the effect of treatment rationales and related strategies on behaviors related to the actual uptake of iCBT. A few studies have examined whether financial incentives (e.g., vouchers, nominal cash payments, or raffles) improve adherence to mental health treatment (32–34), but none have examined their impact on behaviors signaling a willingness to try iCBT. This leaves a notable gap in the literature regarding the potential benefit of providing a small monetary incentive to increase behaviors related to the uptake of iCBT.

The current experimental study examined the effect of a treatment rationale on self-reported acceptability and uptake-related behavior for iCBT among a non-treatment-seeking sample. Acceptability was defined as a set of cognitively based, positive attitudes toward these interventions (17). Given the wide variability in the ways that acceptability has previously been measured, three separate measures were drawn from the literature and analyzed together to measure this construct. The study also examined the effect of a financial incentive ($25 raffle) on seeking information about how to access iCBT programs. Given past research, the authors hypothesized the following: (1) a treatment rationale would increase acceptability for both therapist-assisted and self-guided iCBT, (2) participants would report higher acceptability for therapist-assisted iCBT as compared to self-guided iCBT, and (3) a treatment rationale and a financial incentive would increase behaviors related to the uptake of iCBT.

## Materials and Methods

### Participants

Participants were recruited from a large southeastern University in an urban setting and canvassed from public areas in the surrounding metropolitan area. University student participants (*N* = 403) were recruited online from a University-based research participant pool for psychology course credit. Community participants (*N* = 346) were recruited from public spaces and given the opportunity to enter a raffle with a 1 in 30 chance of winning a $25 gift card as compensation. To be included in the study, participants had to be aged 18 or over and literate in English.

Of the 749 individuals who expressed interest in the study, six respondents were excluded due to failure to meet inclusion criteria. Of the remaining participants (*N* = 743), 81 respondents were excluded from analyses because they took <5 min on the survey or failed the study's manipulation check (11%). In all, data from 662 participants (University *N* = 365; Community *N* = 297) were included for data analysis. Demographic data for these participants are presented in Table 1.

**Table 1:** Participant characteristics.

**Demographics**		**Rationale condition**	**Definition condition**	**Total**
		***N* = 292 (%)**	***N* = 369 (%)**	***N* = 662 (%)**
Age	Mean age (SD)	25.46 (11.88)	25.96 (11.68)	25.76 (11.76)
	Range	18–85	18–73	18–85
Gender	Man	79 (27.1)	149 (40.4)	228 (34.4)
	Woman	208 (71.2)	211 (57.2)	420 (63.4)
	Transgender	1 (0.3)	1 (0.3)	2 (0.3)
	Self-identify	2 (0.7)	8 (2.2)	10 (1.5)
	Did not disclose	2 (0.7)	0 (0)	2 (0.3)
Race/ethnicity	African American/Black	111 (38.0)	148 (40.1)	260 (39.3)
	American Indian or Alaska Native	0 (0)	3 (0.8)	3 (.5)
	Asian American/Asian	37 (12.7)	55 (14.9)	92 (13.9)
	Hispanic/Latino/a	40 (13.7)	41 (11.1)	81 (12.2)
	Multi-racial	16 (5.5)	17 (4.6)	33 (5.0)
	White	82 (28.1)	97 (26.3)	179 (27.0)
	Self-identify	3 (1.0)	8 (2.2)	11 (1.7)
	Did not disclose	3 (1.0)	0 (0)	3 (.5)
Sexual identity	Heterosexual	231 (79.1)	296 (80.2)	528 (79.8)
	Lesbian	4 (1.4)	8 (2.2)	12 (1.8)
	Gay	8 (2.7)	12 (3.3)	20 (3.0)
	Bisexual	27 (9.2)	31 (8.4)	58 (8.8)
	Questioning	12 (4.1)	7 (1.9)	19 (2.9)
	Self-Identify	8 (2.7)	12 (3.3)	20 (3.0)
	Did not disclose	2 (0.7)	3 (0.8)	5 (0.8)
Current financial	Always stressful	21 (7.2)	42 (11.4)	64 (9.7)
Status	Often stressful	67 (22.9)	75 (20.3)	142 (21.5)
	Sometimes stressful	118 (40.4)	164 (44.4)	282 (42.6)
	Rarely stressful	68 (23.3)	67 (18.2)	135 (20.4)
	Never stressful	15 (5.1)	21 (5.7)	36 (5.4)
	Did not disclose	3 (1.0)	0 (0)	3 (0.5)
Treatment history	Received face-to-face psychotherapy	102 (34.9)	145 (39.3)	248 (37.5)
	Has not received face-to-face psychotherapy	185 (63.4)	218 (59.1)	403 (60.9)
	Unsure	0 (0)	1 (0.3)	1 (0.2)
	Did not disclose	5 (1.7)	5 (1.4)	11 (1.5)
	Used an online mental health program	7 (2.4)	7 (1.9)	14 (2.1)
	Did not use an online mental health program	274 (93.8)	354 (95.9)	629 (95.0)
	Unsure	3 (1.0)	1 (0.3)	4 (0.6)
	Did not disclose	8 (2.7)	7 (1.9)	15 (2.3)
Relationship status	Single	171 (58.6)	211 (57.2)	383 (57.9)
	Serious dating or committed relationship	75 (25.7)	97 (26.3)	172 (26.0)
	Civil union, domestic partnership or equivalent	5 (1.7)	3 (0.8)	8 (1.2)
	Married	26 (8.9)	41 (11.1)	67 (10.1)
	Separated	2 (0.7)	5 (1.4)	7 (1.1)
	Divorced	8 (2.7)	10 (2.7)	18 (2.7)
	Widowed	2 (0.7)	0 (0)	2 (0.3)
	Did not disclose	3 (1.0)	2 (0.5)	5 (0.8)

### Procedure

All study procedures were completed using Qualtrics, a survey-creation platform and secure hosting server. University student participants completed the study on their own personal web-enabled devices. Community members completed the study on a tablet computer (i.e., iPad) provided by a research assistant or received an email with instructions to complete the study online.

All participants were assigned a study identification number and completed informed consent procedures prior to starting the study. Upon enrollment, participants were immediately randomized to receive a treatment rationale for iCBT (or a brief definition of iCBT) and a financial incentive to seek information about how to access evidence-based iCBT programs (or none) in a 2 × 2 experimental design. Experimenters were blinded to study condition. Participants first completed questionnaires assessing demographic information and symptoms of depression, anxiety, and stress. Next, depending on experimental condition, participants received a treatment rationale for iCBT or a brief definition of self-guided and therapist-assisted iCBT. Participants then answered questions about their history using and familiarity with online mental health interventions and completed measures of acceptability for self-guided and therapist-assisted iCBT. After completing these measures, participants were informed that they would receive an email within 24 h with a link to access and download iCBT programs, if interested. This link connected participants to a brief online survey in which they could select iCBT programs with empirical support from randomized clinical trials and receive information about how to access them. Depending on experimental condition, participants were also told they would receive a small financial incentive for completing this survey, or not.

### Experimental Conditions

#### Treatment Rationale

Participants assigned to the treatment rationale condition read an in-depth description of iCBT, including rates of usage, research basis, and accessibility. The rationale used persuasion techniques that have been proposed to increase outcome expectancy for psychotherapy, including an authoritative speaker (a University professor and licensed clinical psychologist) and emphasis on empirical support (35). The rationale ended with a “frequently asked questions” section that specifically addressed the most commonly perceived advantages and disadvantages of therapist-assisted iCBT (16). The treatment rationale was ~800 words in length. As a manipulation check, participants who received the treatment rationale then answered three true or false questions about iCBT (see Appendix A.1 for full details).

Participants assigned to the brief definition condition did not receive the treatment rationale. Instead, these participants read a one-paragraph definition of iCBT, which described the difference between self-guided and therapist-assisted iCBT, so that they would have enough information to answer questions assessing their attitudes about these two modalities (see Appendix A.2). The brief definition of iCBT was 130 words in length.

#### Financial Incentive

Participants in the financial incentive condition were offered entry into a raffle with a 1 in 30 chance to a win a $25 e-gift card for completing a survey that included a list of iCBT programs with empirical support from randomized clinical trials about which they would receive information about how to access and download, if interested. Participants assigned to the no financial incentive condition were not offered a financial incentive to complete the survey.

### Measures

#### Demographics and History of Psychotherapy

A 22-item demographics questionnaire was developed for the current study using items from the Standardized Data Set from the Center for Collegiate Mental Health at Penn State University (36). In addition, past and current experience using both face-to-face and Internet-based mental health services was measured using a series of Likert-type self-report items developed for the study (e.g., “Have you ever received face-to-face psychotherapy or counseling?”, “If so, how helpful were these services”).

#### Acceptability of iCBT

##### Attitudes Toward Psychological Online Interventions Scale (APOI)

The APOI is a 16-item validated measure of general attitudes toward online psychological interventions (17). Although many questionnaires have been developed to evaluate acceptability toward Internet-based mental health programs, the APOI is the only psychometrically validated questionnaire to specifically examine this construct. Accordingly, it was selected for the current study over other non-validated questionnaires. Although not indicated in original paper (17), positively valenced items were reverse-coded (Schröder, personal communication, February 12, 2020). Total scores range from 16 to 80 with higher scores indicating more positive attitudes toward iCBT. The APOI demonstrated strong overall internal consistency (α = 0.77) in a sample of 1,013 participants (17) and demonstrated good internal consistency in the present sample for both self-guided iCBT (α = 0.83) and therapist-assisted iCBT (α = 0.82).

##### Credibility/Expectancy Questionnaire (CEQ)

The expectancy subscale of the CEQ (37) consists of 3 items assessing expectations about efficacy for psychological treatments (0–100%), with higher scores indicating higher expectancy of efficacy. It was included in the current study because of its previous use as a measure of iCBT acceptability (18) and to evaluate the effect of outcome expectancy persuasion techniques that were included in the treatment rationale. The CEQ has demonstrated high internal consistency for the overall scale (α = 0.84–0.85), fair to excellent internal consistency for the expectancy subscale (α = 0.79–0.9), and good test-retest reliability [*r* = 0.83; (37)]. The internal consistency of the expectancy subscale in the present sample was excellent for both self-guided iCBT (α = 0.91) and therapist-assisted iCBT (α = 0.90).

##### Single Item

A single Likert scale item assessing willingness to use iCBT, “Would you use a (self-guided/therapist-assisted) iCBT program to improve your life (e.g., reduce stress, anxiety, depression)?” was used as a measure of acceptability based on use of similar items in past research (38). Response choices were scored on a 5-point Likert scale and comprised the following: “definitely would use,” “would likely use,” “unsure,” “unlikely to use,” and “definitely would not use,” with higher scores indicating greater willingness to use iCBT.

#### Psychopathology

##### Depression, Anxiety, and Stress Scale–21 Item (DASS-21)

The DASS-21 is a 21-item validated measure of mental illness symptoms that yields three subscales: depression, anxiety, and stress (39). Scores for the total DASS-21 scale range between 0 and 126, with higher scores indicating more distress or impairment. The DASS-21 demonstrates strong convergent validity with both the Beck Anxiety Inventory (BAI; *r* = 0.81) and Beck Depression Inventory (BDI; *r* = 0.74) indicating satisfactory ability to discriminate between both anxiety and depressive symptoms (40). The DASS-21 demonstrated excellent internal consistency in the present sample (α = 0.90).

### Uptake Behavior for iCBT

Participants were classified as having engaged in behavior related to the uptake of ICBT (or not) if they completed the survey that included a list of iCBT programs about which they would receive information about how to access and download (or not).

### Statistical Analyses

#### Acceptability of iCBT

Age and psychopathology were included as covariates in all models examining acceptability of iCBT, given their association with interest in Internet-based behavioral and psychological treatment (22, 26). A two-way MANCOVA was used to evaluate the effects of rationale condition and recruitment source (community, University) on acceptability of self-guided and therapist-assisted iCBT. A two-way mixed-design MANCOVA was used to evaluate differences in acceptability between self-guided and therapist-assisted iCBT. The three dependent variables within each MANCOVA model included general attitudes (as measured by the APOI), outcome expectancy (as measured by the expectancy subscale of the CEQ), and a single item assessing willingness to use iCBT. For each model, recruitment source was included as an independent variable to test for a two-way interaction. In the absence of an interaction, recruitment source was collapsed and main effects were interpreted across all participants. Listwise deletion was used for participants with missing data, which created variation in sample sizes across models.

#### iCBT Uptake Behavior

A two-step hierarchical logistic regression was performed to test the hypothesis that a treatment rationale (vs. brief definition of iCBT) and a financial incentive (vs. none) would improve participants' likelihood of seeking out information about how to access and download iCBT programs. Uptake behavior was classified as a binary dependent variable (yes vs. no). In step one, four variables were entered to control for participant characteristics that have previously been shown to relate to uptake of iCBT or use of other mental health services: age, psychopathology (DASS-21 total score), gender, and race/ethnicity. Age was included due to evidence that older age is negatively related to use of health-related technologies (41). Psychopathology was included to account for current need for mental health treatment. Race was included due to research showing that African Americans are less likely to initiate iCBT treatment than Whites (42) and U.S. national data demonstrating that White and multiracial individuals seek mental health treatment at higher rates than other racial groups (43). Accordingly, this variable was dummy coded to compare racial identities associated with higher and lower levels of mental health service utilization (White, multiracial vs. Black/African-American, Hispanic/Latinx, Asian). Gender was included due to U.S. national data demonstrating that women seek mental health treatment at higher rates than men (43) and was dummy coded to compare men and women. In step two of the model, treatment rationale and financial incentive conditions were entered to assess the influence of these experimental manipulations while controlling for participant characteristics. All analyses were conducted using Statistical Package for the Social Sciences (SPSS) version 25.0.

## Results

Table 2 shows descriptive statistics and intercorrelations for key variables. Whereas 37.5% of participants reported a history of face-to-face psychotherapy, only 2.1% of participants reported using an online mental health program. Responses to the Depression, Anxiety, and Stress Scale–21 indicated that, on average, participants did not endorse severe levels of psychopathology (*M* = 31.90, SD = 21.80) based on the suggested cutoff of 60 for severe mental illness (39). However, many participants met or exceeded clinical cutoffs suggested by Lovibond and Lovibond (39) for mild depression (Cutoff: 10; *N* = 285, 43.1%), mild anxiety (Cutoff: 8; *N* = 335, 50.6%), or mild stress (Cutoff: 15; *N* = 220, 33.2%), with a total of 411 participants (60.2%) meeting the cutoff for mild symptoms on at least one of these three subscales.

**Table 2:** Means, standard deviations, and correlations between acceptability of iCBT and indicators of mental health symptomatology.

**Variable**	**1**	**2**	**3**	**4**	**5**	**6**	**7**
1. APOI (SG)	1						
2. APOI (TA)	0.72^**^	1					
3. CEQ (SG)	0.46^**^	0.46^**^	1				
4. CEQ (TA)	0.38^**^	0.49^**^	0.86^**^	1			
5. Single Item (SG)	0.45^**^	0.43^**^	0.58^**^	0.49^**^	1		
6. Single Item (TA)	0.31^**^	0.40^**^	0.51^**^	0.54^**^	0.73^**^	1	
7. DASS-21	−0.16^**^	−0.12^**^	0.02	0.03	0.14^**^	0.17^**^	1
*M*	49.37	50.54	12.63	13.57	3.15	3.28	31.90
SD	6.85	6.56	6.75	6.82	1.09	1.08	21.78
Range	16–74	16–80	0–30	0–30	1–5	1–5	0–114

*University Participants (N = 347–363) and Community participants (N = 254–295) depending on the pattern of data missingness. APOI (SG), Attitudes Toward Psychological Online Interventions (Self-guided); APOI (TA), Attitudes Toward Psychological Online Interventions (Therapist-assisted); CEQ (SG), Credibility/Expectancy Questionnaire (Self-guided); CEQ (TA), Credibility/Expectancy Questionnaire (Therapist-assisted); Single Item (SG), Single Item (Would you use Self-guided iCBT to improve your life?); Single Item (TA), Single-Item (Would you use Therapist-assisted iCBT to improve your life?); DASS, Depression, Anxiety, and Stress Scale–21 item*.

** *Significant at p < 0.01*.

### Acceptability of iCBT

#### Assumptions

The three dependent variables within each MANCOVA model were moderately correlated and there was no multicollinearity. Normal distribution of dependent variables was assessed visually and using the Shapiro-Wilk-test. Several dependent variables were significant (*p* < 0.05), however, visual inspection of Q-Q plots revealed that dependent variables were approximately normal. Given that MANCOVA is robust to minor violations of normality (44), the authors proceeded with analyses. Relationships between dependent variables and covariates were linear with homogeneous regression slopes, as determined by visual inspection of scatterplots. Residuals were normally distributed, as assessed by visual inspection of Q-Q plots. To determine the influence of outliers, each model was run with and without univariate and multivariate outliers. All results are reported with outliers included, as removal of outliers did not cause meaningful differences, with one exception (discussed below). Homogeneity of covariance matrices varied across models and is discussed below.

#### Rationale and Self-Guided iCBT

For the two-way MANCOVA (rationale ^*^ recruitment source with age and psychopathology as covariates) examining acceptability for self-guided iCBT, there was homogeneity of covariance matrices, as assessed by Box's *M*-test (*p* = 0.093). The multivariate main effect of recruitment source on the combined dependent variables was significant with seven univariate outliers (standardized residual > 3.0) included in the model (*p* = 0.048), but fell to non-significance when these outliers were removed (*p* = 0.051). Because the outliers appeared to be valid observations, results for this parameter with and without inclusion of outliers are reported.

See Table 3 for multivariate effects. There was no statistically significant interaction effect between rationale condition and recruitment source on the combined dependent variables, *F*_(3,587)_ = 0.762, *p* = 0.516, Wilks' Λ = 0.996, partial η^2^ = 0.004. The main effect of rationale condition on the combined dependent variables was statistically significant, *F*_(3,587)_ = 3.617, *p* = 0.013, Wilks' Λ = 0.982, partial η^2^ = 0.018. There was a statistically significant univariate effect of rationale condition for general attitudes, *F*_(1,589)_ = 9.382, *p* = 0.002, partial η^2^ = 0.016, and for outcome expectancy, *F*_(1,589)_ = 5.886, *p* = 0.016, partial η^2^ = 0.010, such that these two variables were higher for participants who received the treatment rationale. There was no statistically significant univariate effect of rationale condition for the single-item rating of willingness to use iCBT (*p* = 0.133). The main effect of recruitment source on the combined dependent variables was statistically significant with outliers included in the model, *F*_(3,587)_ = 2.657, *p* = 0.048, Wilks' Λ = 0.987, partial η^2^ = 0.013. There was a statistically significant univariate effect of recruitment source on willingness to use iCBT, *F*_(1,589)_ = 7.033, *p* = 0.008, partial η^2^ = 0.012, such that community participants reported greater willingness to use self-guided iCBT. When outliers were removed from this model, the multivariate effect of recruitment source on the combined dependent variables fell to non-significance (*p* = 0.051).

**Table 3:** Multivariate effects for MANCOVA models examining the impact of a treatment rationale on attitudes toward iCBT.

	**Self-guided iCBT**				**Therapist-assisted iCBT**			
	**Wilks' Λ**	***F***	***p***	**Partial **η**^2^**	**Pillai's trace**	***F***	***p***	**Partial **η**^2^**
Age	0.993	1.357	0.255	0.007	0.003	0.636	0.592	0.003
Psychopathology	0.918^*^	17.578	<0.001	0.082	0.068^*^	13.778	<0.001	0.068
Rationale	0.982^*^	3.617	0.013	0.018	0.038^*^	7.421	<0.001	0.038
Recruitment source	0.987^*^^†^	2.657	0.048	0.013	0.010	1.829	0.141	0.010
Rationale × recruitment source	0.996	0.762	0.516	0.004	0.001	0.227	0.878	0.001

* *Significant at p < 0.05*.

† *This effect fell to non-significance (p = 0.051) when outliers were removed from the model*.

#### Rationale and Therapist-Assisted iCBT

For the two-way MANCOVA (rationale ^*^ recruitment source with age and psychopathology as covariates) examining acceptability for therapist-assisted iCBT, Box's *M*-test was significant, indicating a violation of homogeneity of covariance matrices (*p* < 0.001). Accordingly, Pillai's Trace was used as a multivariate test statistic to control for inflation in Type I error rate (45).

See Table 3 for multivariate effects. There was no statistically significant interaction effect between rationale condition and recruitment source on the combined dependent variables, *F*_(3,571)_ = 0.227, *p* = 0.878, Pillai's Trace = 0.001, partial η^2^ = 0.001. The main effect of rationale condition on the combined dependent variables was statistically significant, *F*_(3,571)_ = 7.421, *p* < 0.001, Pillai's Trace = 0.038, partial η^2^ = 0.038. The main effect of recruitment source on the combined dependent variables was not statistically significant, *F*_(3,571)_ = 1.829, *p* = 0.141, Pillai's Trace = 0.010, partial η^2^ = 0.010. There was a statistically significant univariate effect of rationale condition for general attitudes, *F*_(1,573)_ = 12.814, *p* < 0.001, partial η^2^ = 0.022, and outcome expectancy, *F*_(1,573)_ = 6.045, *p* = 0.014, partial η^2^ = 0.010, such that these two variables were higher for participants who received the treatment rationale. There was no statistically significant univariate effect of rationale condition for willingness to use iCBT (*p* = 0.578).

#### Type of iCBT

For the mixed design two-way MANCOVA (type of iCBT ^*^ recruitment source with age and psychopathology as covariates) comparing acceptability for self-guided and therapist-assisted iCBT, there was homogeneity of covariance matrices, as assessed by Box's *M*-test (*p* = 0.053).

See Table 4 for multivariate effects. There was no statistically significant interaction effect between type of iCBT and recruitment source on the combined dependent variables, *F*_(3,558)_ = 0.527, *p* = 0.664, Wilks' Λ = 0.997, partial η^2^ = 0.003. The main effect of type of iCBT on the combined dependent variables was not statistically significant, *F*_(3,558)_ = 2.293, *p* = 0.077, Wilks' Λ = 0.988, partial η^2^ = 0.012. The main effect of recruitment source on the combined dependent variables was statistically significant, *F*_(3,558)_ = 2.650, *p* = 0.048, Wilks' Λ = 0.986, partial η^2^ = 0.014. There was a statistically significant univariate effect of recruitment source on willingness to use iCBT, *F*_(1,560)_ = 7.582, *p* = 0.006, partial η^2^ = 0.013, such that community participants reported greater willingness to use iCBT.

**Table 4:** Multivariate effects for MANCOVA comparing attitudes toward self-guided and therapist-assisted iCBT.

	**Wilks' Λ**	***F***	***p***	**Partial **η**^2^**
Age	0.992	1.465	0.223	0.008
Psychopathology	0.900^*^	20.767	<0.001	0.100
Type of iCBT	0.988	2.293	0.077	0.012
Recruitment source	0.986^*^	2.650	0.048	0.014
Rationale × recruitment source	0.997	0.527	0.664	0.003

* *Significant at p < 0.05*.

### iCBT Uptake Behavior

See Table 5 for results of regression analysis. Participants were excluded from the analysis if they did not fit into the coding scheme for gender (*N* = 13, 2.0%) or race/ethnicity (*N* = 14, 2.1%), did not receive a timely follow-up email with a list of iCBT programs due to experimenter error (*N* = 28, 4.2%), or did not have complete data for variables included in the analysis (*N* = 22, 3.3%). Of the 662 total eligible participants, 588 participants were eligible for regression analysis. Out of these participants, 47 (8.0%) sought out information about how to access and download iCBT programs and 541 (92.0%) did not. Step one of the model, which included participant characteristics, significantly predicted uptake behavior, χ^2^(4) = 12.172, *p* = 0.016. Step one explained 4.8% (Nagelkerke *R*^2^) of the variance in uptake behavior and correctly classified 92.0% of cases, although it should be noted that the model predicted that 100% of participants would not engage in uptake behavior. In this step, psychopathology was positively related to uptake behavior for iCBT (OR = 1.026, *p* = 0.046) and identifying as Black/African-American, Hispanic/Latinx, or Asian was negatively associated with uptake behavior compared to identifying as White or multiracial (OR = 0.509, *p* = 0.029). Step two of the model, which added the rationale and financial incentive conditions as regressors, did not explain significantly greater variability in uptake behavior than step one, Δ*R*^2^ = 0.003, *p* = 0.703. Rationale condition (OR = 0.893, *p* = 0.717) and financial incentive condition (OR = 1.264, *p* = 0.452) did not significantly predict uptake behavior, although the full model remained significant, χ^2^(6) = 12.876, *p* = 0.045.

**Table 5:** Hierarchical logistic regression model predicting uptake-related behavior.

	**Uptake-related behavior**			
	**Step one**		**Step two**	
	***B***	**Odds ratio**	***B***	**Odds ratio**
Constant	−2.920^*^	0.054^*^	−2.952^*^	0.052^*^
Age	0.022	1.022	0.021	1.021
Psychopathology	0.026^*^	1.026^*^	0.025	1.026
Gender	−0.410	0.664	−0.446	0.640
Race/ethnicity	−0.675^*^	0.509^*^	−0.680^*^	0.507^*^
Rationale	-	-	−0.114	0.893
Financial incentive	-	-	0.234	1.264
*R* ^2^	0.048		0.051	
χ^2^	12.172^*^		12.876^*^	
Δ*R*^2^	0.048		0.003	
Δχ^2^	12.172^*^		0.703	

*Significant at p < 0.05.

## Discussion

Consistent with hypotheses, participants who read a treatment rationale reported significant increases in acceptability as measured by general attitudes and outcome expectancy for self-guided and therapist-assisted iCBT across a community and University student sample. Inconsistent with hypotheses, the treatment rationale had no influence on participants' willingness to use either self-guided or therapist-assisted iCBT. Surprisingly, participants' ratings of acceptability (across all three measures) did not significantly differ between self-guided and therapist-assisted iCBT; this finding is inconsistent with prior research, which has generally found that people prefer therapist-assisted over self-guided iCBT. This is the first study to examine the effect of an acceptability-facilitating intervention on behavior related to the uptake of iCBT. Neither the rationale nor the financial incentive influenced uptake behavior, which was very low.

Although the effects of the treatment rationale on acceptability were significant, they were small compared to similar controlled studies of acceptability-facilitating interventions for Internet-based mental health treatment. These interventions, which include treatment rationales, have produced medium-sized increases in acceptability (23, 29). Differences in the effects of acceptability-facilitating interventions between studies may be driven by variations in intervention content, overall length, and method of operationalizing acceptability. For example, past studies examining acceptability-facilitating interventions have used information about iCBT and techniques to increase outcome expectancy, much like in the current study. However, they have also used psychoeducation on specific mental disorders, personalized symptom assessments with feedback, patient testimonials, and appeals to participants' self-efficacy to use a specific program (29, 31, 46).

The smaller effect of the rationale on acceptability of iCBT in the current study relative to past studies may also be related to length. The current study's rationale was ~800 words in length. Previous research has found that treatment rationales of ~250 words may be the optimal length for enhancing outcome expectancy (47). Additionally, Casey et al. (23) found that an acceptability-facilitating intervention of ~400 words caused a medium-sized increase in acceptability for Internet-based mental health treatment. For the current treatment rationale, the authors prioritized describing iCBT in depth, incorporating outcome expectancy persuasion techniques, and addressing perceived advantages and disadvantages of iCBT that have been reported in previous research. The greater length may have caused fatigue or failed to hold participants' attention, which could have prevented participants from fully processing all of the information, thereby reducing its effect. Researchers constructing treatment rationales and other interventions to improve acceptability for iCBT in the future should be aware that acceptability-facilitating interventions which require longer reading times may reduce their impact.

The current study is the first to examine the effects of a treatment rationale and financial incentive on behavior related to the uptake of iCBT. Contrary to hypotheses, neither intervention significantly affected uptake-related behavior. Psychopathology symptom severity and race/ethnicity were associated with uptake of iCBT, although it should be noted that total regression model accounted for a very small proportion of variance (~5%). Participants who reported higher psychopathology were more likely to seek out information about how to access and download iCBT programs. Unlike some prior research on acceptability-facilitating interventions, participants from this study were not drawn from a treatment-seeking sample. It is possible that participants did not believe that they needed iCBT. Lack of a perceived need for mental health treatment is a widely documented barrier to seeking mental health services, particularly among people with mild to moderate symptoms (48). Given that over half of the participants in this study reported at least mild mental health symptoms, interventions designed to increase uptake of iCBT in the general population might have greater success using materials that emphasize the benefit of iCBT as a low-intensity intervention for individuals with mild to moderate symptoms. Personalized feedback about mental health symptoms may be particularly helpful for individuals who are unaware that they may benefit from iCBT. Conversely, people experiencing mild mental health symptoms may believe that they could benefit from iCBT, but be uninterested in making efforts to improve their mental health because their distress is relatively low. Future research on iCBT uptake could evaluate participants' readiness for change and tailor acceptability-facilitating interventions to increase motivation for change if this is a common barrier.

Participants identifying as “White” or “multiracial” were more likely to engage in behavior related to the uptake of iCBT than participants who self-identified as “Black/African-American,” “Hispanic/Latinx,” or “Asian.” Given that people who identify as racial/ethnic minorities are less likely to have access to and to use mental health services (49), this is a sobering finding. Digital mental health interventions, like iCBT, have the potential to overcome practical barriers to mental health treatment that disproportionately affect minority groups, such as cost and transportation (50, 51). The results from this study suggest that these communities may not be inclined to seek out such treatments, simply because they circumvent such practical barriers. Although a small number of studies have examined perceptions and interest in iCBT within specific racial and cultural minority communities (42, 52), many more are needed. It is also critical that future research identify the extent to which acceptability-facilitating interventions need to be culturally tailored to increase uptake in minority communities.

There are meaningful distinctions to be made between general appraisals toward an intervention, personal expectations of efficacy, and a willingness to engage with an intervention—the three dependent measures of acceptability in this study. Our results indicate that interventions which improve general attitudes and outcome expectancy for iCBT programs do not cause corresponding increases in willingness to use them. This may be due to method-variance, given that willingness to use iCBT was assessed using a single item. If, however, the finding is replicated and valid, it is concerning, because it suggests that attitudinal changes caused by treatment rationales and other interventions do not lead to greater self-reported willingness to use iCBT. This is reinforced by this study's finding that the treatment rationale did not increase uptake-related behavior for iCBT. Interestingly, community adults reported slightly greater willingness to use iCBT than University students, an effect that was not attributable to differences in age or psychopathology between samples. This may be due to disparities in access to face-to-face mental health treatment—the University students recruited for this study have access to no-cost counseling services, whereas most community participants likely do not.

### Strengths and Limitations

This is the first experimental study to measure the effects of a treatment rationale on acceptability and uptake-related behavior for iCBT. It is also the first to examine the effect of a financial incentive on uptake-related behavior for iCBT. To date, this is the largest study to examine an acceptability-facilitating intervention for Internet-based mental health treatment. Furthermore, this study operationalized acceptability in a robust way by using three widely used measures of this construct, including a psychometrically validated measure of acceptability toward online mental health interventions. This is important because much of the existing literature that has examined acceptability toward iCBT has used heterogeneous measures of this construct. The need to increase the diversity and inclusion of minority and underrepresented populations in the literature concerning attitudes and utilization of iCBT is paramount. The study used a robust sampling method, recruited a diverse sample of urban community adults and University students, and reported participant characteristics that are associated with underutilization of mental health services. This is a major contribution to the literature; the majority of studies (97%) in a widely cited meta-analysis of randomized controlled trials supporting the efficacy and acceptability of iCBT (19) did not report the racial/ethnic make-up of their sample.

Despite the study's strengths, there are limitations that warrant attention. The small differences between the two rationale conditions in the current study may be due to the nature of the “brief definition” control condition. The authors determined that it was important to define self-guided and therapist-assisted iCBT for participants assigned to the control condition because iCBT is a relatively nascent technology and most people are unfamiliar with Internet-based mental health treatment (14). The brief definition, however, may have functioned like an active control and reduced the comparative effect of the treatment rationale. The use of a survey-based metric for examining uptake-related behavior may have limited our ability to detect true iCBT uptake, as it was insensitive to actual usage of programs. The results for uptake-related behavior cannot be generalized to gender non-conforming people and people outside of the specific racial identities that were predominant in our sample, as we did not have enough of these participants to examine them in our regression model. Additionally, although research has generally supported the use of raffles for incentivizing behavior change, it is possible that the ratio of financial incentive to odds of winning (1:30 chance for $25) was too weak to influence uptake-related behavior. Lastly, the majority of participants were college-educated, which may have implications for measuring attitudes toward Internet-based mental health treatments as educational attainment has been linked to mental health treatment-seeking (53).

### Future Directions

More research is needed to systematically investigate differences in acceptability-facilitating interventions for iCBT that use different types of content. Studies should also examine whether interventions that cause significant improvements in acceptability also lead to measurable increases in uptake-related behavior. Studies examining financial incentives should evaluate the impact of different “doses” of incentive and their cost-effectiveness in healthcare delivery systems. Future research should investigate the relationship between acceptability for iCBT and access to other forms of care across different populations. Studies that recruit diverse samples across different demographic characteristics are vital for understanding the effect of individual characteristics on acceptability and uptake-related behavior for iCBT, as well as other relevant constructs. For example, certain minority racial identities are associated with lower levels of mental health service utilization (43) and racial disparities in trust and experiences with healthcare institutions may play a role in acceptability of digital forms of treatment in comparison to face-to-face care (54). It will be necessary to identify how iCBT appeals differently across racial groups and other demographics to maximize its delivery to those who can most benefit.

### Conclusion

Internet-based cognitive behavioral therapy is well-positioned to leverage its intrinsic benefits of standardization, cost-effectiveness, and ease of access to help fill the gap in unmet mental health need. However, iCBT will be unable to fulfill these goals if acceptability toward these interventions is not significantly improved for the average consumer. The authors hope that future research will build on the findings of the current study to develop effective methods of improving acceptability and uptake-related behavior for iCBT programs in order to fully realize their potential.

## Data Availability Statement

The raw data supporting the conclusions of this article will be made available by the authors, without undue reservation.

## Ethics Statement

The studies involving human participants were reviewed and approved by Georgia State University Institutional Review Board. The patients/participants provided their written informed consent to participate in this study.

## Author Contributions

AM and PA devised the project, the main conceptual ideas, and protocol outline. AM, DE, and LS coordinated data collection for the study and wrote the manuscript. AM conducted all statistical analyses. AM and DE designed the figures and tables. PA supervised the project. All authors contributed to the final version of the manuscript.

## Conflict of Interest

The authors declare that the research was conducted in the absence of any commercial or financial relationships that could be construed as a potential conflict of interest.
