# Supplementary material for: Improving Acceptability and Uptake Behavior for Internet-Based Cognitive-Behavioral Therapy
Source: Front Digit Health. 2021 Mar 25;3:653686. doi: 10.3389/fdgth.2021.653686 (PMC8521972; doi:10.3389/fdgth.2021.653686)
Supplement: Supplementary file 1 [file Data_Sheet_1.pdf]

Improving Acceptability and Uptake Behavior for Internet-Based Cognitive-Behavioral Therapy

Anthony Molloy, Donovan M. Ellis, Langting Su, & Page L. Anderson
Department of Psychology, Georgia State University, Atlanta, GA

Appendix A

Appendix A.1: iCBT Treatment Rationale

Hi, I'm Dr. Anderson!
I'm a professor in the psychology department at Georgia State University.
As a licensed therapist, I've also spent a long time helping people work through common mental health problems like stress, anxiety, and depression.

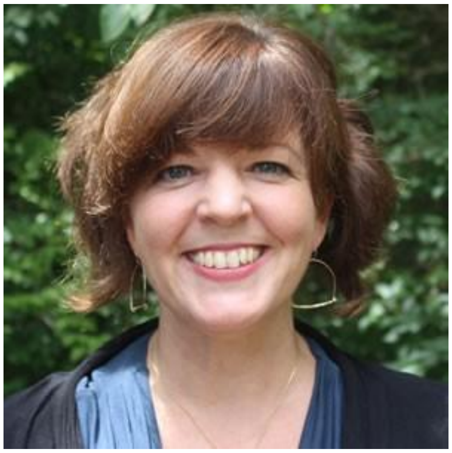

One of my areas of research is online psychotherapy programs, or **iCBT**. The "CBT" stands for cognitive behavioral therapy, which research shows helps people reduce stress, anxiety, and depression. Here's how it works: You work with your therapist to set goals for therapy. CBT works by helping you understand and change thoughts, emotions, and behaviors that are keeping you from reaching your goals for therapy. There is a plan each week for what to work on. CBT works best when you practice the things you learn between therapy sessions, and you and your therapist will decide at the end of each session what you should practice before your next session. CBT is time-limited (typically once a week for about 8 weeks). Traditionally, CBT is done face-to-face, but it can also be done via the internet (iCBT)

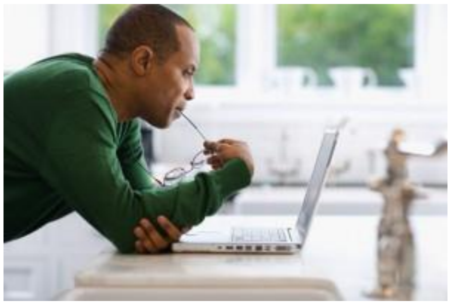

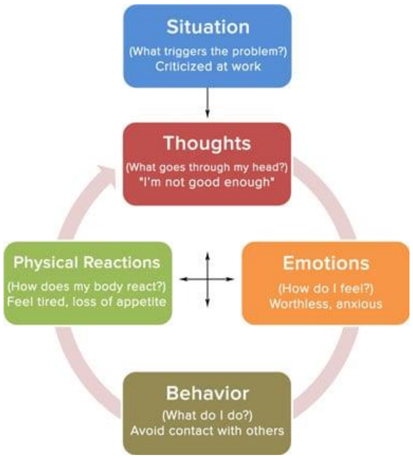

Page Break

iCBT programs are widely used. Millions of people in the U.S. have used online programs and smartphone apps to improve their mental health. These programs are becoming an increasingly integrated part of major healthcare systems.

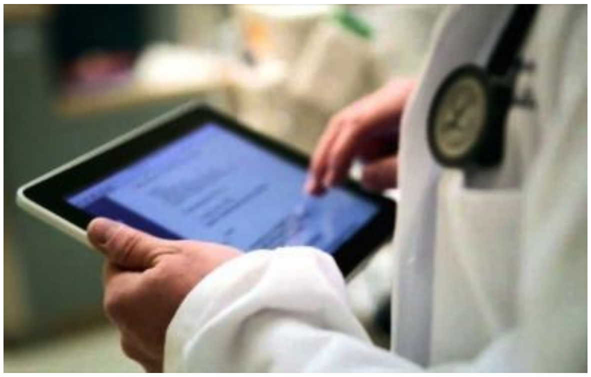

Page Break

It can be intimidating for anyone to find mental health treatment, and especially hard to find the time to meet with someone face to face. That’s one of the major reasons more and more people are deciding to try iCBT programs—you can do them on your own time on your computer or smartphone, so they work on any schedule. In addition to that, the format of CBT is typically easy to deliver online.

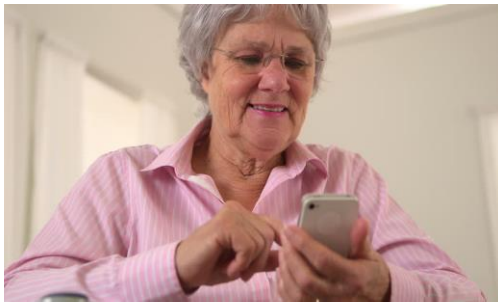

Page Break

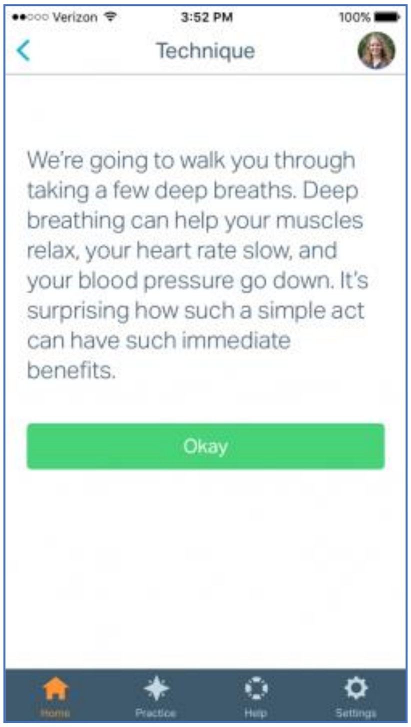

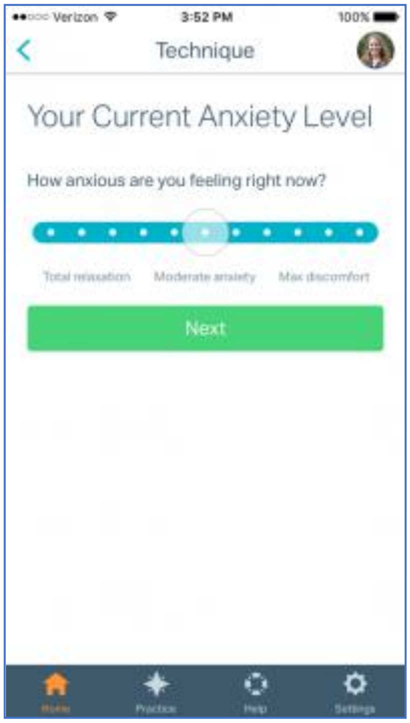

So how does iCBT work?

- Treatment typically involves completing a structured set of lessons online or on a smartphone. These are often done week by week.
- Programs are tailored to specific issues like stress, depression, or anxiety. Some have stories about people overcoming these problems as you gain the tools to do it.
- Lessons usually end with a set of goals to complete before starting the next session. These goals help you put the tools you learn about into action, and might involve something like exercising, introducing yourself to someone new, or keeping a journal of thoughts that cause you distress.

- **Self-guided iCBT** programs are completed on your own at your own pace.

- **Therapist-assisted iCBT** programs involve completing lessons online and working with a therapist via instant messaging, email, phone, or video chat.

Page Break

Frequently Asked Questions

How much time does it take? Can I fit it in my schedule?

Lessons typically take 30 minutes to an hour to complete, and can be completed whenever you have the time. This is one of the major advantages of iCBT. Programs that offer real time interaction with a therapist may involve some scheduling.

How much does it cost?

While cost depends on the program, many of them are free. Some college counseling centers also offer free access to programs.

Is there a waiting period?

You can start most programs right away. Again though, this will depend on the program.

Does it really work?

Over a hundred published studies have shown that iCBT improves stress, anxiety, and depression, among other mental health problems. Most people get relief from symptoms and are highly satisfied with these programs after using them.

Page Break

Frequently Asked Questions

What if I try it and decide I want face to face therapy?

You can always switch. Nothing about starting an iCBT program stops you from seeking in-person therapy. Plus, if your program involves contact with a therapist they might be able to help you find someone.

Will I be able to talk to a therapist?

Some programs are self-guided, while others involve interaction with a therapist via instant messaging, email, phone, or video chat.

What if it's hard for me to write out my problems?

One common worry people have about iCBT is that they're afraid they won't be able to express their thoughts in writing. Most of the self-guided programs don't require writing. Therapist-assisted

iCBT may offer communication through instant messaging, email, phone or video chat. This might be important to consider when looking for a program that works for you.

Is iCBT right for everyone?

iCBT isn't recommended for problems that pose serious risks to your safety. If you've been having thoughts of suicide or feel unsafe in any other way, you should seek in-person help as soon as possible (we'll give you some resources at the end of this survey). Also, some people just prefer talking to a therapist face to face, which is perfectly fine. However, iCBT is a treatment that works well for many people.

Page Break

Thanks for taking the time to learn about iCBT.
I hope the information was useful for you.
When you're ready, click the next button to complete the rest of the survey.

1.) Recap: True or False?

iCBT programs often use lessons, or modules, that can be completed on your own time using a computer or smartphone.

o True

o False

2.) Recap: True or False?

iCBT programs require meeting face to face with a therapist.

o True

o False

3.) Recap: True or False?

Some iCBT programs are completely self-guided, while others involve communication with a therapist via instant messaging, email, phone, or video chat.

o True

o False

Appendix A.2: Brief Definition of iCBT

Online mental health programs directly provide treatment for anxiety, depression, and other mental health problems.

Online cognitive behavioral therapy, or **iCBT** programs, are a common tool for addressing mental health problems. The "CBT" stands for cognitive behavioral therapy, which is a form of psychotherapy that works by helping you understand and change thoughts, emotions, and behaviors. iCBT programs might involve completing a structured set of lessons online. At the end of each lesson, programs often give you goals to practice the things you learn between therapy lessons and based on your feedback will decide which lessons will be completed next, or which may need additional practice for full benefit to you.

Self-guided iCBT programs are done independently.

Therapist-assisted iCBT programs involve support from a therapist via text, email, or videoconferencing.
